# Biological Activity and Metabolomics of *Griffonia simplicifolia* Seeds Extracted with Different Methodologies

**DOI:** 10.3390/antiox12091709

**Published:** 2023-09-01

**Authors:** Giuseppe Mannino, Graziella Serio, Raimondo Gaglio, Massimo E. Maffei, Luca Settanni, Vita Di Stefano, Carla Gentile

**Affiliations:** 1 Plant Physiology Unit, Department of Life Sciences and Systems Biology, University of Turin, Via Quarello 15/A, 10135 Turin, Italy; massimo.maffei@unito.it; 2 Department of Biological, Chemical and Pharmaceutical Sciences and Technologies (STEBICEF), University of Palermo, Viale delle Scienze, 90128 Palermo, Italy; graziella.serio@unipa.it (G.S.); vita.distefano@unipa.it (V.D.S.); 3 Department of Agricultural, Food and Forest Sciences, University of Palermo, Viale delle Scienze, 90128 Palermo, Italy; raimondo.gaglio@unipa.it (R.G.); luca.settanni@unipa.it (L.S.)

**Keywords:** MTT assay, HPLC-MS/MS, Soxhlet, microwave extraction, maceration, 5-hydroxy tryptophan, polyphenols, acetone, ethanol, methanol

## Abstract

*Griffonia simplicifolia*, a tropical plant endemic to West Africa, is highly regarded for its significant pharmacological potential. The objective of this study was to evaluate the metabolomic profile and to explore the antioxidant properties, antiproliferative activity, and antimicrobial potential of *G. simplicifolia* seed extracts obtained through either maceration, microwave-assisted extraction (MAE), or Soxhlet extraction using water, acetone, methanol and ethanol as solvents. Overall, methanol possessed superior total extraction efficiency. HPLC analyses confirmed the efficacy of acetone and ethanol as optimal solvents for the extraction of flavonoids and flavan-3-ols, whereas MAE exhibited enhanced effectiveness in extracting N-containing compounds, including 5-hydroxytryptophan (5-HTP). HPLC-MS analyses identified forty-three compounds, including thirty-four phenolic compounds and nine N-containing molecules. Isomyricitrin, taxifolin and a flavonol glucuronide were the main polyphenols, whereas 5-HTP was the main N-containing compound. Hydroalcoholic *G. simplicifolia* extracts showed the highest radical scavenging and metal-reducing antioxidant power, suggesting that most of the contribution to antioxidant activity depends on the more polar bioactive compounds. *G. simplicifolia* extracts showed dose-dependent antiproliferative activity against three distinct cancer cell lines (HeLa, HepG2, and MCF-7), with notable variations observed among both the different extracts and cell lines and divergent GI_50_ values, emphasizing substantial discrepancies in cell sensitivity to the various extracts. Furthermore, *G. simplicifolia* extracts revealed antibiotic activity against *Staphylococcus aureus*. Our results highlight the potential of *G. simplicifolia* phytochemicals in the development of functional foods, nutraceuticals, and dietary supplements.

## 1. Introduction

Medicinal plants play a crucial role in traditional folk practices, providing effective treatments for a wide range of diseases. Their bioactive molecules can operate both individually or in combination with other substances, demonstrating a peculiar therapeutic potential [1]. Moreover, in comparison to synthetic drugs, natural phytopharmaceuticals have greater acceptance from consumers, improved bioavailability, better safety profiles, and lower toxicity [2].

In recent years, *Griffonia simplicifolia*, a tropical medicinal plant native to West and Central Africa, has received attention because of its bioactive chemicals and possible therapeutic uses. Traditionally, Griffonia is consumed by crafting decoctions or infusions that result in tea-like beverages. Additionally, the seeds can be processed into pastes, extracts, or powders to seamlessly incorporate into various foods and drinks. This botanical marvel boasts a rich heritage in African folk medicine, predominantly attributed to its profound impacts on the central nervous system, mood modulation, and management of gastrointestinal ailments [3,4]. As a result, *G. simplicifolia* and its phytochemicals have been the focus of significant scientific research for the formulation of dietary supplements and/or the creation of new therapeutic medications [5].

Previous phytochemical analyses performed on *G. simplicifolia* showed the presence of a variety of bioactive compounds, and in particular, of non-proteinogenic amino acids. In particular, a plant is a rich source of 5-hydroxytryptophan (5-HTP) [6], a known precursor of serotonin, a neurotransmitter of the human central nervous system [7]. Consequently, *G. simplicifolia* has a potential for use as a therapeutic agent for the treatment of mood disorders, sleep management, and improving cognitive performance, also becoming a good candidate for formulating dietary supplements aimed at preventing mood changes or other serotonin-related disorders [3]. 

*G. simplicifolia* also contains other notable bioactive compounds, such as tannins, flavonols, and carbolide alkaloids [8], which exert a variety of biological actions, including antioxidant, anti-inflammatory, antibacterial, and anticancer activity. Accordingly, the use of *G. simplicifolia* extracts has been investigated for its potential applications in the treatment of migraine, irritable bowel syndrome, fibromyalgia, binge eating, and chronic headache [9].

In this context, extraction of plant phytochemical components is a critical step in unlocking their potential benefits. Indeed, the phytochemical concentration and the potential therapeutic properties of *G. simplicifolia* can be greatly influenced by different extraction procedures [10]. Moreover, a better understanding of the effect of the appropriate extraction procedure of *G. simplicifolia* phytochemicals can improve the bioactive content in the derived formulations by ensuring quality and standardization in medicinal applications. 

To investigate the potential effect of different extraction procedures on *G. simplicifolia* phytochemicals, we analyzed the plant extracts obtained by different extraction methodologies by UV/Vis assay and HPLC-DAD-ESI-MS/MS. Moreover, in order to evaluate the impact of solvent effect, polarity, and extraction techniques on the functional properties of *G. simplicifolia* extracts in terms of antioxidant, antimicrobial and antiproliferative activity, we measured its radical scavenging and metal-reducing activity, evaluated the antiproliferative activity on three different tumoral cell lines (HeLa, HepG2, and MCF-7), and assessed the antibiotic activity on representative Gram-positive and Gram Gram-negative bacteria. 

## 2. Materials and Methods

### 2.1. Plant Material

Seeds of *G. simplicifolia* Baill. were purchased from BioResources International Inc., (Lafayette, LA, USA) and obtained directly from farmers in Ghana. After the seeds were received, they were stored at room temperature until the next analysis was started. Seeds were grinded before extraction, finely sieved and then used for the various extraction processes.

### 2.2. Preparation of Extracts

#### 2.2.1. Soxhlet Extraction (SE)

The dried powder of *G. simplicifolia* seeds was extracted using a Soxhlet apparatus as previously described [11]. In brief, the heating mantle was set to 70 °C, and the extraction was carried out using a 1:10 (*w*/*v*) ratio with three different solvents (methanol, ethanol, or acetone) for 300 min. Following the extraction processes, the extracts were then filtered through a Millex HV 0.45 μm filter (Millipore, Billerica, MA, USA) and stored at −80 °C until analysis. While most of the resulting extract was stored at −80 °C until further analysis, a representative aliquot was first evaporated under vacuum and then dried in an oven at 50 °C overnight. The dried powder was then weighed to calculate the extraction yield.

#### 2.2.2. Maceration (ME)

Dried *G. simplicifolia* powdered seeds were placed in an orbital shaker with a solvent by using a 1:10 (*w*/*v*) ratio and macerated for 60 h at room temperature in the dark. Three different solvents (methanol, ethanol or acetone) were used for maceration. Additionally, three different solvent/water ratios were used ([95% (*v*/*v*), 70% (*v*/*v*), and 50% (*v*/*v*))]. After 3 days, the macerates were centrifuged (10 min at 10,000× *g*, 4 °C), and the supernatant was removed, stored at 4 °C and replaced with an equal volume of pure solvent. After 24 h, the supernatants were combined and filtered through a Millex HV 0.45 μm filter (Millipore, Billerica, MA) and stored at −80 °C until analysis. As for SE, while most of the resulting extract was stored at −80 °C until further analysis, a representative aliquot of all macerates was evaporated under vacuum and then dried in an oven at 50 °C overnight. The dried powder was then weighed and the extraction yield was calculated.

#### 2.2.3. Microwave-Assisted Extraction (MAE)

*G. simplicifolia* was extracted using a microwave by combining the methods published by Tao-Xie [12] and Tarin [13]. Briefly, MAE was conducted by placing a flask containing the dried seed powder in a microwave oven (MCG-LG, MG583, Mumbai, India) set at 100 W. A 1:10 (*w*/*v*) ratio with distilled water was used. The extraction was carried out for 10 min, with interruptions every 2 min for 30 s to prevent the mixture from boiling. After MAE, the extract was centrifuged (10 min at 10,000× *g*, 4 °C) and filtered through a Millex HV 0.45 μm filter (Millipore, Billerica, MA, USA). After lyophilization, an aliquot of MAE was dried in an oven at 50 °C overnight and weighed to calculate the extraction yield.

### 2.3. Total Polyphenol Content (TPC)

The total polyphenol content (TPC) was quantified as previously described [14]. Briefly, 20 μL of each extract were incubated with an equal amount of Folin–Ciocâlteu reagent, 10 μL of 20% (*w*/*v*) Na_2_CO_3_ and 150 μL of deionized water (dH_2_O) in a 96-well plate. Samples were then left at 80 °C for 1 min and at 25 °C for 25 min. Finally, the absorbance was measured at 725 nm in a microplate reader (NB-12-0035, Neo Biotech, Nanterre, France). TPC was expressed as mg of gallic acid (VWR International, Radnor, PA, USA) equivalents (GAE) 100 g^−1^ fresh weight (FW) by plotting the sample absorbance versus serial dilution of pure standard. All measurements were performed in three different biological replicates.

### 2.4. Total Proanthocyanidin Content (TPAC)

The total proanthocyanidin content (TPAC) was evaluated by incubating 28 μL of each sample with 84 μL 0.1% (*w*/*V*) 4-(dimethylamino)cinnamaldehyde (DMAC) solution, composed of 75% *(v*/*v*) ethanol and 12.5% HCl. The results were expressed as mg A2-type PAC (Extrasynthese^®^, Genay, France) equivalents (PACE) 100 g^−1^ FW by plotting the sample absorbance versus the serial dilutions of pure standard [15]. All measurements were performed in three different biological replicates.

### 2.5. Total Alkaloid Content (TAC)

The total amount of alkaloids in the different extracts was evaluated via the spectrophotometric method described by Singht and co-workers [16]. Briefly, 1 mL of a mixture containing 25 mM FeCl_3_ and 0.5 M HCl was added to the flask in which appropriate dilutions of the *G. simplicifolia* extracts were previously pipetted. Then, 1 mL of 50 mM 1,10-phenanthroline dissolved in ethanol was added; distilled water was used to reach 10 mL. Each flask was placed in a water bath maintained at 70 °C for 30 min. Finally, the absorbance of the red-colored products was measured at 510 nm against a blank that was prepared in the same experimental condition, but where the diluted sample was replaced by distilled water. Quantification was performed using an external calibration curve of atropine (10–100 μg mL^−1^), and data were expressed as mean ± standard deviation (SD) of three different replicates.

### 2.6. Antioxidant Activity

#### 2.6.1. ABTS Assay

The ABTS assay is based on the ability of antioxidants contained in *G. simplicifolia* extracts to scavenge the 2,2′-azino-bis(3-ethylbenzothiazoline-6-sulfonic acid) (ABTS) radical cation (ABTS^+^). ABTS^+^ was generated by incubating 7 mM ABTS with 2.45 mM potassium persulfate for 16 h in the dark and at room temperature. After ABTS^+^ formation, the solution was diluted until an absorbance value of about 0.70 was recorded at 734 nm. Subsequently, 20 μL of appropriately diluted samples were combined with 180 μL of ABTS^+^ in a 96-well plate. Following a 5 min incubation period, the reduction in color of the resulting mixture was assessed by measuring the absorbance at the identical wavelength [14]. A calibration curve of 6-hydroxy-2,5,7,8-tetramethylchroman-2-carboxylic acid (Trolox) (VWR International, Milan, Italy) was employed to determine the radical scavenging activity, and expressing data as mmol of Trolox equivalent (TE) 100 g^−1^ of FW of fresh weight (FW). Each measurement was replicated thrice.

#### 2.6.2. DPPH Assay

The DPPH assay is based on the reduction of the stable free radical DPPH by antioxidants [17]. Briefly, 0.1 mM 1,1-diphenyl-2-(2,4,6-trinitrophenyl)hydrazine (DPPH) solution was diluted until an absorbance value of about 0.90 was monitored at 517 nm. Consequently, 190 µL of the diluted DPPH mixture was added to 10 µL of properly diluted extract. After 20 min, the absorbance was read at 517 nm against a reaction incubated with ethanol as a blank. An external calibration curve of Trolox was used to estimate the radical scavenging activity, and the results were expressed as mmol TE 100 g^−1^ FW. All measurements were repeated three times.

#### 2.6.3. FRAP Assay

The FRAP assay measures the ability of an antioxidant to reduce ferric ions (Fe^3+^) to ferrous ions (Fe^2+^). This reduction is monitored by measuring the change in absorbance at 595 nm after incubating a properly diluted sample with 300 mM of sodium acetate buffer (pH = 3) containing 2.5 mM FeCl_3_ and 1.25 mM 2,4,6-Tripyridyl-s-triazine (TPTZ) [17]. After the incubation time, the absorbance of each well was read at 595 nm against a blank achieved by the incubation of the assay buffer with pure ethanol. Trolox was used as a reference standard, and the metal-reducing antioxidant power was expressed as mmol TE 100 g of FW. The experiments were repeated three times.

### 2.7. HPLC-DAD-ESI-MS/MS

The HPLC system consisted of an Agilent Technologies 1200 connected to a DAD and a 6330 Series Ion Trap LC-MS System (Agilent Technologies, Santa Clara, CA, USA) coupled with an electrospray ionization (ESI) source. The chromatographic separation was performed at a constant flow rate of 0.2 mL min^−1^ using a reverse phase C18 Luna column (3.00 μm, 150 mm × 3.0 mm i.d., Phenomenex, Torrance, CA, USA) maintained at 25 °C by an Agilent 1100 HPLC G1316A Column Compartment. 

#### 2.7.1. Alkaloids and N-Containing Compounds

For alkaloid analysis, we used a binary solvent system, using MilliQ H_2_O acidified with 0.1% *v*/*v* formic acid (Solvent A) and acetonitrile acidified with 0.1% *v*/*v* formic acid (Solvent B). During the chromatographic run, N-containing compounds were separated using an isocratic elution of 97% A and 3% B. To ensure column cleanliness before the subsequent injection, the concentration of Solvent B was gradually increased and maintained at 3% for 5 min. Each sample injection volume was 5 μL. UV–VIS spectra were recorded between 220 and 800 nm, and chromatographic profiles were registered at 230 and 260 nm. Tandem mass spectrometry analyses were conducted in positive mode, with a nitrogen flow rate of 5.0 mL min^−1^ maintained at 325 °C. The capillary voltage was set at 1.5 kV. Due to the impossibility of commercially available standards of pure molecules, N-containing compounds were identified using a semi-quantitative approach, as previously reported [11].

#### 2.7.2. Flavonoids 

To analyze flavonoids, we utilized a binary solvent system. Solvent A consisted of MilliQ H_2_O acidified with 0.1% (*v*/*v*) formic acid, while Solvent B consisted of acetonitrile acidified with 0.1% (*v*/*v*) formic acid. The initial solvent composition was 90% (*v*/*v*) A and 10% (*v*/*v*) B for 5 min. Subsequently, the proportion of Solvent B was increased to 55% (*v*/*v*) A over 25 min, followed by a final concentration of 70% (*v*/*v*) B in 25 min. At the end of each chromatographic run, the initial solvent composition was restored and maintained for an additional 10 min before the next injection. The sample injection volume for each analysis was 10 μL. UV/Vis spectra were recorded in the range of 220–650 nm, and the chromatographic profiles were monitored at 280, 360, and 520 nm. For the identification and quantification of flavonoids, tandem mass spectrometry analyses were conducted in negative mode, as previously described [18].

### 2.8. Evaluation of Antiproliferative Activity

#### 2.8.1. Cell Culture 

HeLa (human cervical cancer), HepG2 (human hepatocarcinoma) and MCF-7 (human breast adenocarcinoma) cells were obtained from the American Type Culture Collection (Rockville, MD, USA) and cultured in Roswell Park Memorial Institute (RPMI) as previously described [14]. Before performing experiments, the cells were trypsinized approximately between 75–85% of their confluence, and exponentially growing cells were used for the evaluation of antiproliferative activity.

#### 2.8.2. MTT Assay 

The 3-[4,5-dimethylthiazole-2-yl]-2,5-diphenyltetrazolium bromide (MTT) assay was performed as previously described [19]. In brief, cells were seeded into 96-well plates and incubated for 24 h. Subsequently, each *G. simplicifolia* extract was appropriately diluted and added to the cells, followed by another 48 h incubation. Tested concentrations of each extract were freshly prepared by dilutions of the extract in the complete culture medium. In each experiment, the solvent (methanol, ethanol or acetone) concentration never exceeded 0.25%, and a culture medium with 0.25% methanol, ethanol, or acetone was used as a control. After the incubation time, a medium containing 0.5 mg mL^−1^ of MTT reagent was introduced to each well and discarded after 3 h incubation at 37 °C. Consequently, cells were lysed, and the produced formazan salt was dissolved using dimethyl sulfoxide (DMSO). The absorbance of the MTT–formazan complex was measured at 570 nm using a microplate reader (GloMax^®^ Multidetection Plate Reader, Promega^®^). The percentage of growth (PG) in relation to untreated cells (control) was calculated using Equation 1.

PG (%) = [(OD_test_ − OD_tzero_)/(OD_ctr_ − OD_tzero_)] × 100 (1)

where OD_test_ was the optical density (OD) measurements before exposure of cells to the test extract, and OD_tzero_ was the measurement after the incubation time. Finally, OD_ctr_ was the measurement after the same incubation time but with no cell exposure to the different treatments. The concentration necessary for 50% of growth inhibition (GI_50_) for each extract was calculated by plotting concentration−response curves. Each result is a mean value of five separate experiments.

### 2.9. Evaluation of Antibacterial Activity

#### 2.9.1. Bacterial Strains

Two bacterial strains belonging to the American Type Culture Collection (ATCC) were used as indicator microorganisms (sensitive to antimicrobial compounds) towards *G. simplicifolia* seed extracts. To this purpose, *Escherichia coli* ATCC25922 among Gram-negative and *Staphylococcus aureus* ATCC33862 among Gram-positive bacteria were chosen as representative of the main food-borne bacterial pathogens. These bacteria were propagated twice in Nutrient Broth (NB) (Condalab, Madrid, Spain) and incubated at 37 °C for 24 h [20].

#### 2.9.2. Evaluation of Antibacterial Properties

*G. simplicifolia* extracts were evaluated in vitro for antibacterial properties, as previously reported [21]. Briefly, 2% (*w*/*v*) agar water plates were overlaid with 7 mL of 0.7 (*w*/*v*) NB soft agar medium, previously inoculated with 10^7^ CFU mL^−1^ of each indicator strain. After soft agar solidification, sterile paper discs (Whatman^®^ qualitative filter paper, Grade 1) of 0.6 cm diameter were placed onto the surface of the double layer agar and soaked with 10 µL of each *G. simplicifolia* seed extract, a negative control mixture (acetone, ethanol or methanol) or a positive control (10% *w*/*v* streptomycin). The negative control mixtures were properly diluted in sterile water until the antimicrobial activity of the solvents could be undetectable [18,22]. The same dilution factor was then applied to *G. simplicifolia* extracts [18,22]. Antibacterial activity was assessed by measuring the diameter of the clear area around the paper discs with a digital caliper (Stainless Hardened, Farnell, Kraków, Poland). The test was carried out in triplicate.

### 2.10. Statistical Analysis

All results were expressed as mean ± standard deviation (SD). ANOVA followed by Tukey’s post-hoc test was applied, when possible, to all data obtained from chemical and cellular quantifications. A *p* ≤ 0.05 value was used as a criterion of significance. All statistical analyses were carried out using SPSS Statistics 24 (SPSS, Chicago, IL, USA). Heat maps analysis coupled with cluster analysis were generated using the average linkage and Pearson distance methods.

## 3. Results and Discussion

### 3.1. Extraction Yield of G. simplicifolia Seeds Depends on Solvent Polarity and Water Content 

**Figure 1:**
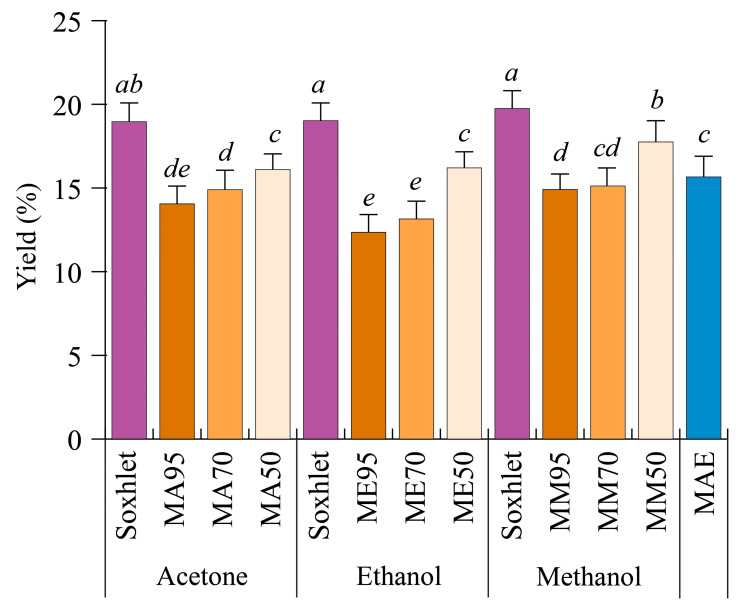
Extraction yields of *Griffonia simplicifolia* seeds based on different extraction methods and solvents. MA, maceration with acetone; ME, maceration with ethanol; MM, maceration with methanol. The percentage of the solvents with respect to water during maceration are 50, 70 and 95. MAE, microwave-assisted extraction. Lowercase letters on the top of the bars indicate statistical differences (*p* < 0.05) as measured by one-way ANOVA followed by Tukey’s post hoc test.

In order to evaluate whether different techniques affect the extraction yield of *G. simplicifolia* seeds, the plant’s raw material was extracted continuously by either Soxhlet, maceration, or MAE methods. Soxhlet extraction was performed using either pure acetone, methanol, or ethanol, whereas maceration was carried by 95% (*v*/*v*) (M95), 70% (*v*/*v*) (M70), and 50% (*v*/*v*) (M50) mixtures of the same solvents and water. Finally, MAE was performed for 10 min using only water as the extraction solvent. Figure 1 reports the different yields obtained by the extraction methods used.

In general, the extraction yields ranged from 12.35% ± 1.20% (ME95) to 19.75% ± 1.06% (Soxhlet with methanol, SM). Regardless of the solvent employed for the extraction process, yields obtained with the Soxhlet method were always higher (19% *w*/*w*, *p* < 0.05) than those obtained after maceration. In the latter, yield differences depended not only on the type of solvent used but also on the water content. Indeed, the use of pure solvents resulted in lower yields, while increasing the percentage of water led to higher yields (Figure 1). As a general yield trend, a M50 > M70 > M95 decreasing trend was observed, with methanol and acetone being the best extraction solvents. MAE showed average yield values when compared to the other methods (Figure 1).

Plant constituents of different species can be extracted efficiently by using solvents with different polarities. In comparative studies, methanol proved to be highly efficient for the extraction of bioactive compounds present in several plant species, with regards to other organic solvents [23,24], whereas the comparison between acetone and ethanol indicates acetone as the best solvent [25,26]. With regards to MAE, although inferior to Soxhlet or maceration with 50% water, it can be considered a fast method for the extraction of molecules from *G. simplicifolia* seeds. When different extraction methods were evaluated to recover bioactive compounds from rosemary leaves, MAE allowed comparable yields in a shorter time, with the advantages of MAE in preserving some easily degradable compounds [27]. Similar results have also been described for grape pomace [28], *Lotus* leaves [29], *Eucalyptus* leaves [30], and *Ginkgo* leaves [31]. Therefore, our results suggest that MAE could be a suitable and sustainable method for the extraction of some *G. simplicifolia* seed phytochemicals.

### 3.2. Acetone and Ethanol Are the Best Solvents for Flavonoids and Flavan-3-ols, whereas MAE Is More Efficient for N-Containing Compounds

**Figure 2:**
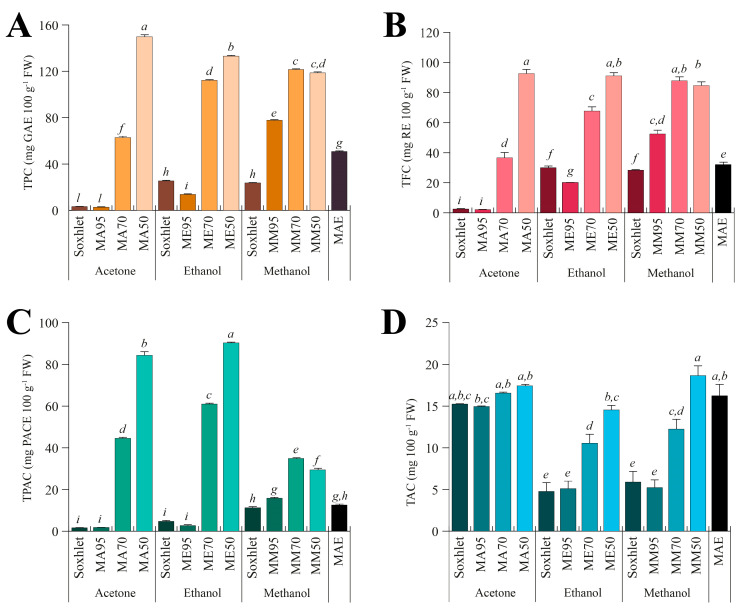
Total content of polyphenols (TPC, (**A**)), flavonoids (TFC, (**B**)), proanthocyanidins (TPAC, (**C**)), and alkaloids (TAC, (**D**)) measured in the different seed extracts of *G. simplicifolia*. Plots report the content of bioactive components according to the extraction technique (Soxhlet, maceration, or microwave-assisted), the type of solvent used (acetone, ethanol, or methanol), and the amount of water added for maceration (M95, M70, or M50). MAE, microwave-assisted extraction. Bars represent the content expressed as mg 100 g^−1^ fresh weight (FW), and the metric bars are the standard deviation. Lowercase letters above each bar indicate statistical differences (*p* < 0.05) as assessed by one-way ANOVA followed by Tukey’s post hoc.

To evaluate the chemical composition of the different extracts, the total phenolic content (TPC, Figure 2A), total flavonoid content (TFC, Figure 2B), total proanthocyanidin content (TPAC, Figure 2C), and total alkaloid content (TAC Figure 2D) were assessed.

Regarding the TPC (Figure 2A), a consistent difference was observed among solvents, with values ranging from 2.73 ± 0.11 (MA95) to 149.88 ± 3.14 (MA50) mg GAE 100 g^−1^ FW. MA50 exhibited the highest TPC content, followed by ME50, MM70 and MM50. Acetone is a less polar solvent compared to methanol and ethanol. Consequently, it is conceivable that phenolic compounds present in *G. simplicifolia* have a greater affinity for the more polar alcoholic solvents, resulting in an increase in TPC with increasing water content. This is also consistent with the low TPC content of Soxhlet extracts, where pure solvents were used. Similar results have been previously obtained on the TPC from various plant species, including *Ziziphus mucronata* stem bark [32], *Senna italica* leaves [33], almonds [34], walnuts [35], and several wild edible legumes [36]. Specifically, in these studies, it was observed that extraction with pure methanol and ethanol produced higher TPC values than the extraction with acetone.

A similar trend was observed when analyzing the TFC (Figure 2B) of the different *G. simplicifolia* extracts, while some small differences were observed for the TPAC (Figure 2C). Specifically, TFC exhibited a very similar pattern to TPC; whereas, although the trend of TPAC broadly resembles that observed in TPC and TFC, significantly (*p* < 0.05) higher values were observed for both acetone and ethanol extracts, while methanol extracts showed the lowest values (Figure 2C). 

The higher extraction efficiency of acetone towards PACs is already known in the literature. PACs are complex molecules derived from the polymerization of various flavan-3-ol subunits and show poor polarity when the polymerization degree is high. Therefore, the use of less polar solvents, such as acetone, aids in the extraction process of high molecular weight PACs [37]. Additionally, acetone is more capable of establishing hydrogen bonding, π-π stacking, and hydrophobic interactions with PACs, which are almost absent in alcoholic solvents [38]. These interactions facilitate acetone to preferentially extract PACs over other components of the plant matrix, resulting in higher yields of these compounds. According to this result, in a study by Ramos et al. (2020), the extraction of PACs from pine bark was evaluated using different solvents, including acetone, ethanol, and methanol. The authors showed that extraction with acetone produced higher levels of proanthocyanidins, especially those with a higher molecular weight, compared to extraction with methanol. This indicates that acetone was more effective in extracting larger and more complex proanthocyanidin polymers [39]. Under our experimental conditions, this finding may also suggest the presence of high molecular weight PACs in *G. simplicifolia* extracts.

Finally, for TAC (Figure 2D), regardless of the extraction method and water percentage, the series of extracts obtained using acetone showed the highest values, while the series in methanol recorded the lowest values. In contrast to TPC, TFC and TPAC, the extract obtained with MAE using water as extraction solvent recorded high values (Figure 2D). Some alkaloids can be extracted more effectively with MAE in water than with traditional extraction methods using organic solvents due to the baric and thermal increase generated by microwave energy [40]. Moreover, MAE hydrothermal extraction has been found to provide the highest yield of total alkaloids when compared to other solvents [37].

### 3.3. Isomyricitrin, Taxifolin and a Flavonol Glucuronide Are the Main Polyphenols of G. simplicifolia

**Figure 3:**
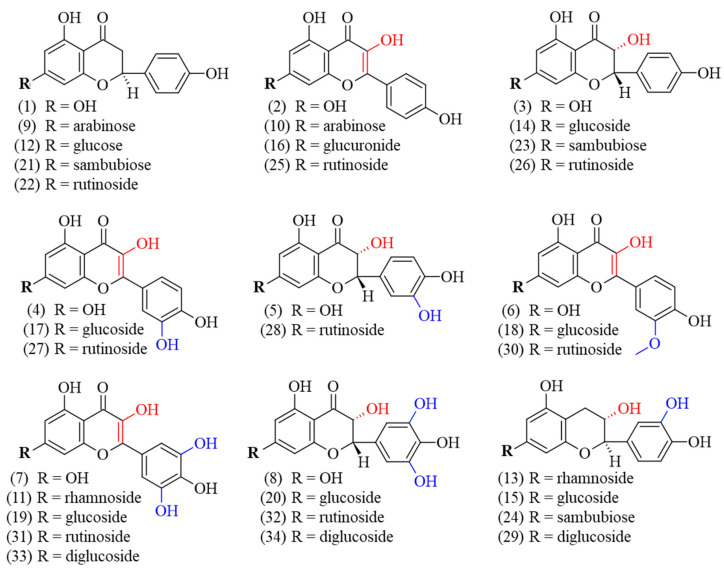
Chemical structure of the identified polyphenolic compounds of *G. simplicifolia* extracts. Formulae numbers refer to the compounds cited in the main text or in Supplementary Table S1. The red color indicates the position of hydroxyl groups bound to the C ring of the flavonoid scaffold, while the blue color indicates the hydroxyl groups linked to the B ring of the same chemical scaffold.

Currently, the literature on *G. simplicifolia* has primarily focused on the content of specific N-containing compounds, and in particular 5-HTP, due to its potential therapeutic applications [6]. As a result, limited data are currently available on the specific identification and characterization of flavonoids and other classes of plant secondary metabolites. By using HPLC coupled to MS/MS, we identified and quantified diverse bioactive molecules in the different *G. simplicifolia* extracts. Overall, we identified 43 compounds, including 34 phenolic compounds (Figure 3) and 9 N-containing molecules.

The identified polyphenols were widely distributed among the different extracts and belonged to several classes. Specifically, twelve flavonols (kaempferol (**2**), quercetin (**4**), myricetin (**7**), juglanin (**10**), myricitrin (**11**), kaempferol–glucuronide (**16**), isoquercitrin (**17**), isomyricitrin (**19**), kaempferol–rutinoside (**25**), rutin (**27**), myricetin–rutinoside (**31**), and myricetin–diglucoside (**33**)), ten flavanols (aromadendrin (**3**), taxifolin (**5**), ampelopsin (**8**), aromadendrin–glucoside (**14**), ampelopsin–glucoside (**20**), aromadendrin–sambubioside (**23**), aromadendrin–rutinoside (**26**), taxifolin–rutinoside (**28**), ampelopsin–rutinoside (**32**), and ampelopsin–diglucoside (**34**)), five flavanones (naringenin (**1**), naringenin–arabinoside (**9**), prunin (**12**), naringenin–sambubioside (**21**), and naringenin–rutinoside (**22**)), four flavan-3-ols (catechin–rhamnoside (**13**), catechin–glucoside (**15**), catechin–sambubioside (**24**), catechin–diglucoside (**29**)), and three 3-methylated flavonols (isorhamnetin (**6**), isorhamnetin-glucoside (**18**), and narcissin (**30**)) were identified. Most of the phenolic compounds were conjugated to sugars, while only eight were aglycones (**1**, **2**, **3**, **4**, **5**, **6**, **7**, and **8**). Concerning the glycosylated polyphenols, fifteen were monoglycosides, with two being conjugated to arabinose (**9** and **10**), seven to glucose (**15**, **12**, **14**, **20**, **17**, **19**, and **18**), two to rhamnose (**11** and **13**), one to glucuronic acid (**16**), and three to sambubiose (**21**, **23** and **24**). In addition, eight polyphenols were conjugated with rutinoside (**22**, **26**, **28**, **32**, **25**, **27**, **31**, and **30**) and three were diglucosides (**29**, **33** and **34**). Figure 3 shows the chemical structures of the identified polyphenols.

**Figure 4:**
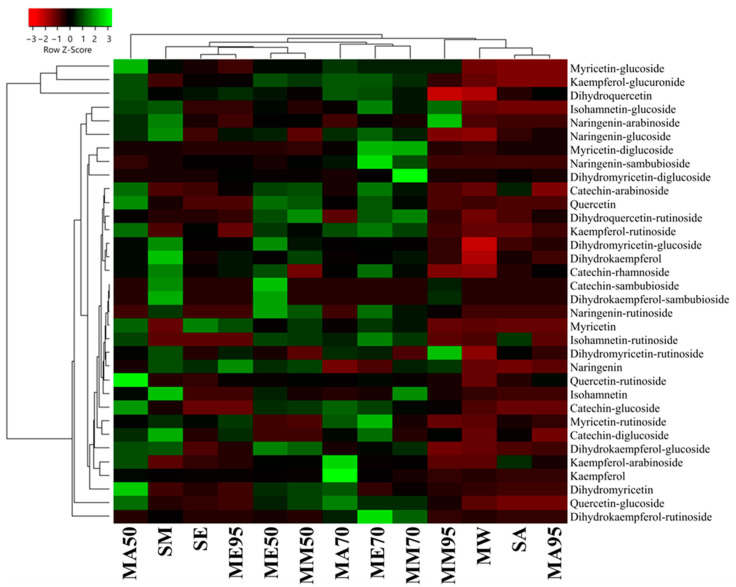
Heatmap coupled to cluster analysis showing the polyphenolic compound variation among the different extracts of *G. simplicifolia*. Quantitative data are supported in Supplementary Table S1. The dendrogram was generated using average linkage and Pearson distance methods; the different colors refer to the highest (green) or lowest (red) relative amounts of molecules among the analyzed extracts.

From a quantitative point of view, the polyphenol amount measured using HPLC-DAD-ESI-MS/MS revealed that MAE had the lowest amount of polyphenols (26.25 ± 0.74 μg 100 g^−1^ FW), followed by SA (64.11 ± 1.85 μg 100 g^−1^ FW ), MA95 (61.25 ± 0.98 μg 100 g^−1^ FW), and MM95 (61.25 ± 0.98 μg 100 g^−1^ FW) (Figure 4, also see Supplementary Table S1 for raw quantitative data). ME95 also showed a low polyphenol content (137.22 ± 2.23 μg 100 g^−1^ FW). On the other hand, the best yields were obtained for macerates, specifically, those in which the extraction solvent was aqueous mixtures consisting of 50% (*v*/*v*) or 70% (*v*/*v*) organic solvent, as shown by their portioning in a separate cluster (Figure 4). Specifically, these macerates show a median value of phenolic compounds of 273.23 ± 4.58 μg 100 g^−1^ FW. Although MAE exhibited lower quantitative performance, it was the only extract in which all 34 phenolic compounds were detected. Furthermore, MAE is based on the sole use of water as the extraction solvent. This not only makes it a rapid extraction method (10 min) but also increases its sustainability and minimizes environmental risks by ensuring a safer method of extraction.

When comparing the conventional extraction methods (maceration and Soxhlet apparatus), methanol confirmed to be the most effective solvent for polyphenols extraction (with a median value of 223.22 ± 17.24 μg 100 g^−1^ FW), followed by acetone (211.08 ± 22.85 μg 100 g^−1^ FW) and ethanol (202.44 ± 6.78 μg 100 g^−1^ FW). In terms of polyphenol content, the acetone series demonstrated the highest levels in the order MA50 > MA70 > MA95. On the other hand, the alcoholic (methanol and ethanol) macerates displayed the highest polyphenol content in the 70% (*v*/*v*) series, while the lowest levels were observed in the 95% (*v*/*v*) series. Finally, comparing SA, SE, and SM with their respective MA95, ME95, and MM95, no substantial differences (*p* > 0.05) were observed, either from a quantitative or a qualitative point of view.

Among all the analyzed extracts, compounds **5**, **16**, and **19** exhibited the highest abundance regardless of the extraction method used, comprising approximately 40–60% of the total identified polyphenols. However, their concentrations were significantly higher in the extracts obtained through conventional methods compared to MAE.

Interestingly, although the DMAC assay successfully detected the presence of PACs, HPLC analysis allowed for the identification and quantification of simple flavan-3-ols, whether in a glycone form or conjugated with sugars. However, it is important to note that the DMAC assay is well known to exclusively react with flavan-3-ol polymers (namely PACs), regardless of their degree of polymerization. In contrast, the assay does not react with simple flavan-3-ols [41]. Therefore, due to the limitations of our analytical method (HPLC with a MW limit of 2600 *m*/*z*), the absence of PACs in the HPLC analysis does not exclude the possibility that PACs having a high polymerization degree may actually be present. This information, in combination with the DMAC analysis, suggests that PACs are present in the various extracts of *G. simplicifolia*, but their degree of polymerization is significantly higher than what can be analyzed using our HPLC-ESI-MS/MS system.

### 3.4. 5-HTP Is the Most Important N-Containing Compoud of G. simplicifolia and Is Best Extracted by Organic Solvent Maceration in the Presence of Water

**Figure 5:**
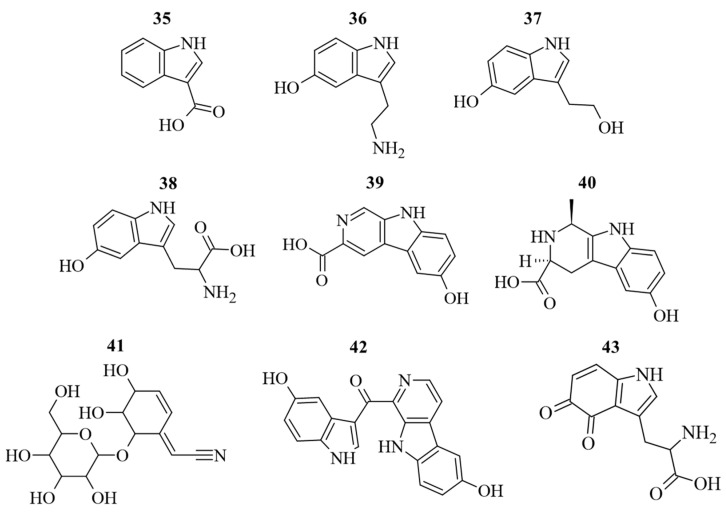
Chemical structure of the identified alkaloid compounds in *G. simplicifolia* extracts. Formulae numbers refer to the compounds cited in the main text and in Supplementary Table S2.

Along to polyphenols, also N-containing compounds were detected and quantified (Figure 5). The identification and quantification of N-containing compounds in *G. simplicifolia* extracts play a crucial role in understanding the potential health benefits of this plant material. One of the key components responsible for its beneficial effects is 5-HTP (**38**), a well-known precursor of serotonin and then melatonin [9]. In our previous study, we used HPLC-DAD-ESI-MS/MS to profile dried *G. simplicifolia* extracts, with a specific focus on identifying and quantifying **38** [11]. In the current analysis, we identified **38**, along with other N-containing compounds, including two tryptamines (5-hydroxytryptamine (**36**) and 5-hydroxy-3-(2-hydroxyethyl)-indole (**37**)), a β-carboline (3-carboxy-6-hydroxy-β-carboline (**39**)), a pyridoacridine (hyrtioerectine B (**40**)), an iridoid glycoside (griffonin (**41**)) and a pyridoacridine (hyrtiosulawesine (**42**)). Moreover, in addition to **38**, our analysis revealed the presence of two other compounds containing an indole scaffold (1H-indole-3-carboxylic acid (**35**) and tryptophan-4,5-dione (**43**)). In agreement with our previous analyses [11], compounds **35**–**43** were detected in all extracts obtained from *G. simplicifolia*. Figure 5 depicts the chemical structure of N-containing bioactive compounds of *G. simplicifolia*.

In addition, our HPLC-DAD-ESI-MS/MS analyses report the complete absence of 1,1′-ethylidenebis(l-tryptophan) and 3-(phenylamino)alanine, also known as peak E and peak UV-5, respectively. Previous studies have correlated the presence of peak E [42] and peak UV-5 [43] to processes involving genetically engineered bacteria during fermentation. In addition to the potential risks related to undesirable microbiological contamination, it has been shown how peak E and peak UV-5 induce functional activation of human eosinophils and interleukin 5 production from T lymphocytes, resulting in the onset of neurological diseases of high severity, including eosinophilia–myalgia syndrome [44,45,46]. Our results confirm that *G. simplicifolia* is unable to produce peaks E and UV-5, which remain important markers of 5-HTP production via transgenic bacterial fermentation.

**Figure 6:**
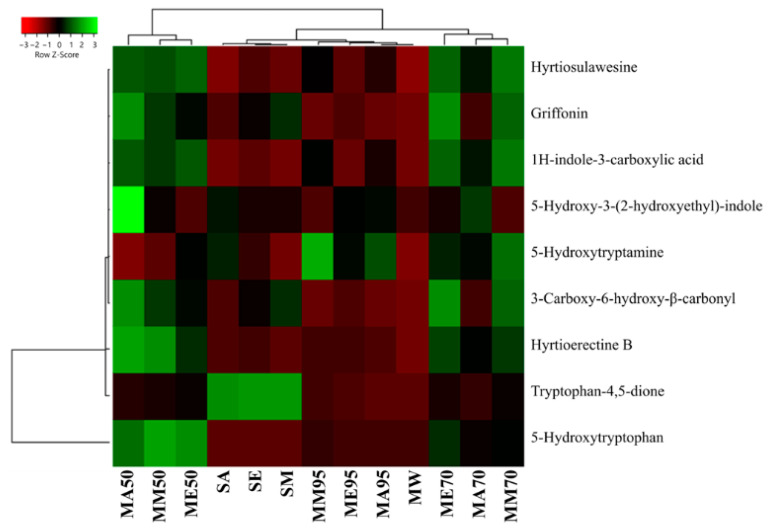
Heatmap coupled to cluster analysis showing the alkaloid compound variation among the different extracts of *G. simplicifolia*. Quantitative data are supported in Supplementary Table S2. The dendrogram was generated using average linkage and Pearson distance methods; the different colors refer to the highest (green) or lowest (red) relative amounts of molecules among the analyzed extracts.

From a quantitative perspective, the lowest extraction yield for compound **38** was found with the Soxhlet method, both in terms of absolute amount (ranging from 0.65 ± 0.02 mg g^−1^ FW, in acetone extracts, to 2.41 ± 0.08 mg g^−1^ FW, in methanolic extracts) and as a ratio calculated considering its relative degradation product (**43**), also known as peak X_1_ [47] (Figure 6, also see Supplementary Table S2 for raw data). Concerning **43**, it can naturally originate from plants through the conversion of the amino acid tryptophan via the kynurenine pathway. In this pathway, the indole amino acid is first converted into N-formylkynurenine by the enzyme tryptophan 2,3-dioxygenase (EC 1.13.11.11) and then transformed into **43** [48]. However, it has also been observed that its production can depend on the excessive oxidation of **38** during specific redox reactions [49,50]. Aside from the interesting biological functions of **43** as a metabolic intermediate for quinolinic acid production or as a neurotransmitter modulator acting as an antagonist of glutamate receptors, the dione has also demonstrated alarming excitotoxicity. Indeed, overactivation of excitatory neurotransmitters, such as glutamate, can contribute to neuronal cell damage and death [51,52]. For this reason, other than monitoring peak E and peak UV-5 content in bacterial production of 5-HTP, it is important that peak-X_1_ (**43**) amounts remain low, especially when *G. simplicifolia* is marketed as a dry extract [47]. Our analyses revealed that all solvent extracts have an approximately 3-fold higher content of **43** than **38**, while in macerates and MAE, the content is lower than 2 mg g^−1^ FW. Moreover, for these extracts, the content of **38** was from 25-fold to 90-fold higher than **43** (Figure 6, Supplementary Table S2). 

The increase of **43** in the Soxhlet extracts may be the consequence of excessive heating during the extraction process, which inevitably results in the oxidation of many bioactive molecules. This hypothesis is further confirmed by the high levels of **38** and low amounts of **43** that have been measured in MA95, ME95, and MM95, in which the same pure solvents were used without the aid of high temperatures. Consequently, the best extraction methodology for **38** from *G. simplicifolia* is maceration using 50% (*v*/*v*) ethanol or methanol, which resulted in the extraction of 149.21 ± 2.92 mg g^−1^ FW and 133.71 ± 1.15 mg g^−1^ FW, respectively. Moreover, the same extracts recorded very low levels of **43**.

Moreover, for the other N-containing compounds, the macerates using hydroalcoholic solvents proved to be the most efficient extraction method. In particular, MA50 had the highest yields (8.51 ± 0.24 mg g^−1^ FW), whereas, in MA95, ME95 and MM95 yields were the lowest, reporting a comparable content of approximately 2.30 mg g^−1^ FW.

### 3.5. Hydroalcoholic Extracts Show the Highest Radical Scavenging and Metal-Reducing Antioxidant Power 

The intrinsic antioxidant activity of a plant matrix depends on the quantity and type of phytochemicals present and is commonly referred to as total antioxidant activity (TAA) [53]. Phytochemicals with antioxidant properties exert their activity primarily through two mechanisms: (i) hydrogen atom transfer (HAT) or (ii) single electron transfer (SET) [54]. In both cases, the process leads to the neutralization of free radicals and the oxidation of antioxidant species. During the HAT reaction, the antioxidant donates a hydrogen atom to the free radical, resulting in the oxidation of the antioxidant to a stable non-radical species. On the other hand, in the SET reaction, after the transfer of an electron to the free radical, it converts the antioxidant into a non-reactive cationic radical, which is resonance-stabilized [55]. Consequently, a single assay may not be sufficient to reliably evaluate the total antioxidant activity of a sample. For this reason, different assays using various reactive species and different mechanisms for quenching reactive species provide more reliable estimates of the antioxidant potential of the sample while also providing valuable information about the mechanism involved in the execution of the reducing activity. Based on these considerations, in this study, the total antioxidant activity of *G. simplicifolia* seed extracts was determined using ABTS, DPPH, and FRAP. The DPPH and ABTS assays measure the radical scavenging activity of the sample by utilizing reactive radical species, while the FRAP assay measures its metal-reducing activity (Table 1).

For acetone extracts, the antioxidant activity values varied widely for each of the performed assays. Regardless of the assay, the antioxidant activity values increased as the percentage concentration of acetone decreased (Table 1). The lowest values were observed for MA95, while the highest values were observed for MA50. The only exception was the FRAP value of MA70, which was slightly higher than that recorded for MA50. SA exhibited TAA that was almost equivalent to those recorded for MA95, which had the lowest antioxidant activity values. 

Significant variability was also observed for ethanol extracts. Similar to what was observed for acetone macerates, TAA increased as the percentage concentration of the solvent decreased. However, unlike the acetone extracts, the antioxidant activity values for SE were slightly higher than those recorded for ME95, and the ABTS values were significantly lower (Table 1).

Moreover, for methanol extracts, values varied within a wide range for each of the considered assays. Regarding the effect of the percentage of organic solvents on the antioxidant activity of the maceration extracts, significant differences were observed depending on the assay used to measure the TAA. Specifically, higher ABTS values were recorded for MM50 and MM70 compared to MM95, while the lowest antioxidant activity was measured for SM. The DPPH value of MM50 was approximately double compared to MM70 or MM95. On the other hand, the FRAP assay recorded lower values that were not statistically different regardless of the methanol content. For SM, MM95 also recorded very low values when antioxidant activity was measured via ABTS and DPPH assays. Conversely, SM recorded the highest FRAP value (Table 1). 

The differences in absolute values of antioxidant activity measured with the different assays may depend on the variations in the characteristic electron transfer mechanism of each assay, as well as the characteristics of the reaction mixtures and the ability of the antioxidant components of the extract to interact with the reactive species characteristic of each assay. Despite these possible differences, our analyses show both a good correlation between the antioxidant activity assays used (ρ > 0.75) and that TAA increases with increasing water content in the extraction solvent, suggesting that most of the contribution to antioxidant activity depends on the more polar bioactive compounds.

### 3.6. G. simplicifolia Extracts Exhibit a Concentration-Dependent Antiproliferative Activity, with a Significant Variability across the Different Cell Lines

The antiproliferative activity of *G. simplicifolia* seed extracts was evaluated by MTT assay against three human epithelial cancer cell lines (HeLa, HepG2 and MCF-7). Taking into consideration both the amount of gastric juices (about 600 mL) [56] and the dosage commonly used for *G. simplicifolia* supplement formulation, we selected an experimental range between 200 and 2000 μg DW mL^−1^ of cell medium. Accordingly, under our experimental conditions, the highest tested concentration was achievable in 1 mL of gastrointestinal fluids after ingestion of commercially available *G. simplicifolia* supplements. 

The extracts exhibited a concentration-dependent antiproliferative activity, with significant variability across the different tumor cell lines. Furthermore, GI_50_ values with respect to each cell line highlight significant differences in cell sensitivity as a function of the extraction protocol (Table 2).

While a strong correlation between the GI_50_ values of HeLa and HepG2 cell lines (ρ = 0.966) was observed, indicating similar cellular sensitivity to the tested extracts, the GI_50_ values of HeLa and HepG2 cells and those of MCF-7 cells were poorly correlated (ρ < 0.50) (Table 2). Specific protein expression profiles of MCF-7 cells could explain a peculiar response to extracts with distinct phytochemical compositions [57,58].

Specifically, for acetone extracts, the GI_50_ values, expressed as µg d.wt. mL^−1^ cell medium, ranged from 1141.97 ± 80.33 to 1189.55 ± 66.35 for the HeLa cell line, and from 1076.74 ± 73.22 to 1152.66 ± 58.44 and from 922.96 ± 73.68 to 1332.55 ± 89.56 for the HepG2 and MCF-7 cell line, respectively (Table 2). 

Acetone extracts demonstrated higher inhibitory activity against the MCF-7 cell line, while HeLa and HepG2 cell lines showed lower sensitivity. While MA50 and MA70 showed comparable activity on HeLa and HepG2 cells, MA50 was more active than MA70 on MCF-7 cells. On the contrary, the extracts with the highest percentage of acetone, MA95 and Soxhlet, were not active in the tested concentration range.

Ethanol and methanol extracts were more active than those in acetone (Table 2). Concerning ethanol extracts, the highest activity was recorded for the extract at a 70% (*v*/*v*) solvent/water ratio. On the other hand, for the extracts in acetone, and also for those in methanol, Soxhlet and the extract at a 95% (*v*/*v*) solvent/water ratio (ME95) did not exhibit activity within the tested concentration range. On the other hand, a good activity for all methanol extracts, including Soxhlet and MM95, on HepG2 and HeLa cell lines was recorded without significant differences in the GI_50_ values, with an average value below 800 µg d.wt. weight mL^−1^ cell medium. On the contrary, MCF-7 cell sensitivity to ethanol extracts depended on the extraction protocol. In this case, the most active extracts were Soxhlet and ME70. Concerning HeLa and HepG2 cell lines, we found a good correlation between TPC values and GI50 values, while antiproliferative activity is poorly related to antioxidant activity (ρ < 0.50). 

The biological activity of phytochemicals has more frequently been related to their redox-active properties; with small changes in the cellular redox balance, phytochemicals are able to affect the structure and function of several redox-sensitive biological targets [59]. However, increasing experimental evidence suggests that the binding ability of these small molecules to specific biological targets could justify the observed bioactivity without involving the antioxidant mechanism [60]. Here, the distinct dissimilarities in antiproliferative activity among *G. simplicifolia* seed extracts, along with the low correlation with radical scavenging and reducing activity, encourage deep investigations into the interaction between their phytochemical compositions and biological effects. 

Recent single-molecule studies have shed light on the specific *G. simplicifolia* phytochemical components responsible for this activity, revealing their modulation of critical pathways such as cell cycle progression and induction of apoptosis [61]. In this context, the wide range of bioactive compounds present in *G. simplicoflia* extracts, such as flavonoids, alkaloids, and other N-containing compounds, are well known to interact with cellular processes, exerting an inhibitory influence on uncontrolled cell growth. For example, certain alkaloids have demonstrated the ability to arrest cell cycle progression at specific checkpoints [8], while flavonoids demonstrate pro-apoptotic effects through modulation of apoptotic signaling pathways [62]. Nonetheless, while the individual compounds may possess intrinsic inhibitory potential, their synergistic action might exert amplified efficacy, underscoring the holistic nature of *G. simplicifolia* antiproliferative activity. To the authors’ knowledge, there are currently no scientific studies investigating the antiproliferative activities of *G. simplicifolia*, and even less evidence demonstrating the antiproliferative effects of purified fractions or compounds from *G. simplicifolia* seeds on tumoral human cell lines. Consequently, further investigations are needed to better understand the mechanism of action underlying the observed biological effects.

### 3.7. Preliminary Tests Show That G. simplicifolia Seed Extracts Possess Antimicrobial Activity against *S. aureus*

The antibacterial properties of *G. simplicifolia* seed extracts were tested against two representative pathogenic bacteria (*E. coli* and *S. aureus*), responsible for food-borne illnesses [63], by means of the disc diffusion method. This technique is commonly used to classify bacterial strains as resistant or susceptible to natural antimicrobial extracts [64]. Indeed, the higher the clear area around the paper discs, the more susceptible the bacterial strains to plant extract [65]. To our knowledge, no previous work evaluated the antibacterial activity of *G. simplicifolia* seed extracts against either Gram-negative or Gram-positive bacteria. So far, the antibacterial activity of *G. simplicifolia* has been known only in relation to leaf extracts, showing efficacy against Gram-positive but also Gram-negative bacteria and against some fungi [62]. Here, for the first time, we evaluate the antibacterial activity of *G. simplicifolia* seeds against *E. coli* ATCC25922 and *Staphylococcus aureus* ATCC33862 in order to expand our understanding of the potential antibacterial properties of this plant species beyond its traditionally studied leaf extracts. The results of the inhibitory activity of the *G. simplicifolia* seed extracts are reported in Table 3. 

The dilutions of acetone, ethanol or methanol using sterile water and employed as negative controls did not reveal any antibacterial activity against the two pathogenic bacteria used as indicators of microorganisms; this finding confirms what has been previously reported [66]. Moreover, the extract obtained by the microwave-assisted extraction technique did not reveal any antibacterial effect against the tested strains. Teng and Lee (2014) assessed that the absence of antimicrobial activity in the microwave-assisted plant-derived extracts is undoubtedly due to the limited time of this extraction process [67]. Effectively, although MAE is a rapid and safe method for the extraction of bioactive components from plant matrices, our HPLC analysis revealed that the extract obtained through this technique appears to have a lower content of bioactive compounds than the others.

All *G. simplicifolia* seed extracts, regardless of the solvent employed for the extraction process, were not able to inhibit the growth of *E. coli*. This result is mainly imputable to the presence of a lipopolysaccharide covering layer in the external membrane of Gram-negative bacteria, which is characterized by low permeability [68]. Therefore, this structure acts as a barrier to increase the resistance of Gram-negative bacteria to the plant-derived antimicrobial compounds [69]. Regarding Gram-positive bacteria, *S. aureus* was sensible to the Soxhlet and macerates extracts of *G. simplicifolia* seeds. In particular, the antibacterial activities of ethanol, methanol and acetone extracts obtained by Soxhlet were 18.2, 17.5 and 16.9 mm, respectively, while the macerate extracts were recorded at about 2 mm lower in M95 and 5 mm in M70. These results are not surprising because the Soxhlet technique is characterized by a shorter extraction time and a higher temperature than the maceration procedure; thus, the former technique promotes a more consistent extraction of antimicrobial components [70]. However, the antibacterial activity of these extracts against *S. aureus* is mainly imputable to their interaction with the peptidoglycan of the cell wall that destabilizes bacterial permeability [71]. Our data showed that seed extracts of *G. simplicifolia* show potential for the treatment of Gram-positive bacterial infections. However, due to the limited use of bacterial strains in this study, further analyses are needed to ascertain the broader spectrum of antibacterial activity exhibited by *G. simplicifolia* seeds. Comprehensive investigations involving a wider array of bacterial species, along with variations in extraction techniques and concentrations, will provide a more comprehensive understanding of the seeds’ potential as a versatile antibacterial agent. Such expanded research will assess the feasibility of incorporating *G. simplicifolia* seeds into diverse antimicrobial applications.

## 4. Conclusions

This study provided valuable insights into the bioactivity of *G. simplicifolia* seed extracts and highlighted the importance of extraction methods to optimize their phytochemical composition and, consequently, their biological activity. Through a comparative analysis, we found that even though the extraction methods significantly affected the metabolic profile of *G. simplicifolia* seed extracts, methanol was the best solvent to achieve the highest yield. However, acetone and ethanol proved to be the best solvents for flavonoids and flavan-3-ols extraction, whereas MAE was more suitable for the extraction of N-containing compounds, including the bioactive 5-HTP. *G. simplicifolia* seed extracts showed a significant antioxidant capacity, suggesting their ability to reduce oxidative stress and defend against cellular damage, particularly for hydroalcoholic extracts. In addition, by studying the antiproliferative activity of the different extracts on three different cancer cell lines (HeLa, HepG2 and MCF-7), a concentration-dependent antiproliferative activity was observed, with significant variability across both the different extracts and cell lines. Finally, the antimicrobial activity of *G. simplicifolia* extracts showed significant antimicrobial effects against *S. aureus*. However, the cellular mechanism underlying the observed biological effects deserves further investigation. Additional studies are currently underway to improve their comprehensive characterization, and results will be reported soon.

The results of this work highlight the bioactive potential of *G. simplicifolia* seed extracts. The identified compounds of *G. simplicifolia* seeds exert antioxidant properties, potential antitumor properties and antibiotic effects. Overall, our results can contribute to (i) support the optimization of the extraction process, (ii) aid the selection of appropriate extraction methods for specific applications, and (iii) ensure the maximum utilization of *G. simplicifolia* constituents for the development of new drugs and dietary supplements.

## Figures and Tables

**Table 1:** Radical scavenging activity (ABTS and DPPH) and ferric reducing antioxidant power (FRAP) of *G. simplicifolia* seed extracts. The antioxidant activity is reported as mmol of Trolox Equivalent 100 g^−1^ d.wt. Values are expressed as mean ± SD of three experiments carried out in triplicate.

		DPPH	ABTS	FRAP
**Acetone**	Soxhlet	0.201 (0.001) ^g^	0.196 (0.004) ^l^	0.003 (0.001) ^i^
	MA95	0.197 (0.003) ^g^	0.151 (0.001) ^l^	0.025 (0.001) ^i^
	MA70	0.749 (0.023) ^e^	8.884 (0.217) ^f^	1.775 (0.055) ^c^
	MA50	1.568 (0.036) ^d^	22.493 (0.146) ^b^	1.153 (0.023) ^d^
**Ethanol**	Soxhlet	0.618 (0.020) ^ef^	4.482 (0.049) ^h^	0.421 (0.010) ^f^
	ME95	0.373 (0.035) ^fd^	10.646 (0.196) ^e^	0.255 (0.005) ^g^
	ME70	3.811 (0.157) ^b^	19.889 (0.882) ^d^	3.111 (0.032) ^b^
	ME50	5.187 (0.356) ^a^	22.561 (0.265) ^b^	4.568 (0.047) ^a^
**Methanol**	Soxhlet	0.318 (0.023) ^fg^	3.753 (0.025) ^h^	0.553 (0.008) ^e^
	MM95	1.956 (0.075) ^c^	2.297 (0.058) ^i^	0.317 (0.006) ^g^
	MM70	1.923 (0.014) ^c^	25.088 (0.218) ^a^	0.275 (0.008) ^g^
	MM50	3.824 (0.097) ^b^	21.235 (0.244) ^c^	0.259 (0.011) ^g^
**MAE**		0.145 (0.011) ^g^	5.987 (0.448) ^g^	0.102 (0.002) ^h^

Different letters indicate significant (*p* < 0.05) differences. For abbreviations, see Figure 1 and Figure 2.

**Table 2:** Antiproliferative activity of *G. simplicifolia* seed extracts against HeLa, HepG2 and MCF-7 tumor cell lines. Cell growth was measured by MTT assay after 48 h of treatment with different concentrations of each extract and was expressed as GI_50_ (µg of d.wt. weight per mL of cell medium). Data represent mean ± SD of three independent experiments. Among the same series (HeLa, HepG2 or MCF-7), different letters indicate significant differences at *p* ≤ 0.05, as measured by Tukey’s multiple range test. Letter “a” denotes the highest antiproliferative activity.

		HeLa	HepG2	MCF-7
**Acetone**	**Soxhlet**	>2000	>2000	>2000
	**MA95**	>2000	>2000	>2000
	**MA70**	1141.97 (35.7) ^a^	1076.74 (42.16) ^a^	1332.55 (33.01) ^a^
	**MA50**	1189.55 (47.43) ^a^	1152.66 (37.77) ^a^	922.96 (35.26) ^b^
**Ethanol**	**Soxhlet**	>2000	>2000	>2000
	**ME95**	>2000	>2000	>2000
	**ME70**	784.6 (10.55) ^b^	634.32 (7.74) ^c^	760.5 (20.26) ^c^
	**ME50**	1042.43 (11.53) ^a^	792.4 (22.13) ^b^	789.4 (18.21) ^c^
**Methanol**	**Soxhlet**	649.88 (13.82) ^c^	809.52 (11.22) ^b^	701.84 (11.06) ^d^
	**MM95**	693.45 (22.68) ^c^	809.65 (25.03) ^b^	1029.25 (34.72) ^e^
	**MM70**	603.15 (21.08) ^c^	803.69 (23.88) ^b^	719.59 (22.49) ^cd^
	**MM50**	1072.89 (30.66) ^a^	777.16 (24.01) ^b^	1460.73 (39.11) ^a^
**MAE**		1580.32 (37.27) ^d^	1744.3 (40.34) ^d^	1654.32 (48.62) ^f^

**Table 3:** Antibacterial activity of *G. simplicifolia* seed extracts. Results indicate the mean values ± standard deviation (S.D.) of three assays.

Solvents	Samples	Inhibition (mm)	
		*E. coli* ATCC25922	*S. aureus* ATCC33862
Acetone	Soxhlet	n.d.	16.9 ± 0.2
	MA50	n.d.	n.d.
	MA70	n.d.	11.6 ± 0.1
	MA95	n.d.	14.4 ± 0.2
		n.d.	
Ethanol	Soxhlet	n.d.	18.2 ± 0.3
	ME50	n.d.	n.d.
	ME70	n.d.	13.3 ± 0.2
	ME95	n.d.	16.1 ± 0.4
		n.d.	
Methanol	Soxhlet	n.d.	17.5 ± 0.3
	MM50	n.d.	n.d.
	MM70	n.d.	12.3 ± 0.2
	MM95	n.d.	15.8 ± 0.4

*E. coli* = *Escherichia coli*; *S. aureus* = *Staphylococcus aureus*; n.d. = not detectable.

## Data Availability

All of the data is contained within the article and the supplementary materials.

## Supplementary Materials

The following supporting information can be downloaded at https://www.mdpi.com/article/10.3390/antiox12091709/s1, Table S1: Qualitative and quantitative chemical analysis of polyphenolic compounds extracted from *G. simplicifolia* seeds identified by HPLC–ESI-MS/MS analysis; Table S2: Qualitative and quantitative chemical analysis of alkaloid compounds extracted from *G. simplicifolia* seeds identified by HPLC–ESI-MS/MS analysis.
