# Supplementary material for: Biological Activity and Metabolomics of Griffonia simplicifolia Seeds Extracted with Different Methodologies
Source: Antioxidants (Basel). 2023 Sep 1;12(9):1709. doi: 10.3390/antiox12091709 (PMC10525635; doi:10.3390/antiox12091709)
Supplement: Supplementary file 1 [file antioxidants-12-01709-s001.zip › antioxidants-2573899-supplementary.pdf]

Supporting Table 1: Qualitative and quantitative chemical analysis of polyphenolic compounds extracted from *G. simplicifolia* seeds identified by HPLC–ESI-MS/MS analysis. Table reports Molecular Weight (MW), ionized mass (m/z), chemical name and quantification in the different extracts. Data are expressed as µg per 100 g of FW.

#	MW	m/z		Soxhlet			Acetone Maceration			Ethanol Maceration			Methanol Maceration			MAE
				Acetone	Ethanol	Methanol	50% (v/v)	70% (v/v)	95% (v/v)	50% (v/v)	70% (v/v)	95% (v/v)	50% (v/v)	70% (v/v)	95% (v/v)	
**1**	272.25	271.25	*Naringenin*	n.d.	1.644 (0.023)	1.992 (0.075)	0.9 (0.021)	n.d.	0.249 (0.007)	1.665 (0.032)	0.41 (0.006)	2.729 (0.091)	1.865 (0.062)	1.54 (0.018)	1.773 (0.083)	0.158 (0.001)
**2**	286.23	285.23	*Kaempferol*	n.d.	1.455 (0.042)	1.999 (0.09)	3.281 (0.114)	17.043 (0.569)	0.186 (0.003)	2.13 (0.061)	2.323 (0.075)	1.79 (0.064)	1.79 (0.023)	1.201 (0.017)	n.d.	0.413 (0.006)
**3**	288.25	287.25	*Dihydrokaempferol*	2.356 (0.054)	2.365 (0.099)	5.966 (0.279)	3.05 (0.123)	2.578 (0.068)	1.874 (0.05)	2.634 (0.042)	2.735 (0.105)	3.163 (0.064)	3.811 (0.105)	3.28 (0.126)	1.947 (0.019)	0.337 (0.008)
**4**	302.23	301.23	*Quercetin*	n.d.	n.d.	1.107 (0.02)	5.82 (0.219)	1.858 (0.076)	n.d.	5.023 (0.064)	4.339 (0.091)	n.d.	4.529 (0.156)	2.277 (0.101)	n.d.	0.392 (0.011)
**5**	304.25	303.25	*Dihydroquercetin*	26.156 (0.442)	38.911 (0.639)	33.675 (0.644)	49.317 (2.128)	49.797 (1.001)	33.22 (1.064)	37.663 (0.44)	47.861 (0.667)	43.074 (0.717)	29.35 (0.753)	37.657 (1.179)	39.12 (1.01)	4.049 (0.081)
**6**	316.26	315.26	*Isohamnetin*	n.d.	n.d.	5.12 (0.134)	1.339 (0.057)	0.304 (0.008)	n.d.	1.878 (0.075)	0.628 (0.029)	n.d.	0.542 (0.011)	4.083 (0.114)	0.488 (0.008)	0.147 (0.001)
**7**	318.23	317.23	*Myricetin*	n.d.	0.898 (0.03)	n.d.	0.781 (0.027)	0.318 (0.003)	n.d.	0.346 (0.01)	0.6 (0.008)	0.684 (0.018)	0.608 (0.006)	0.492 (0.014)	n.d.	0.042 (0.001)
**8**	320.25	319.25	*Dihydromyricetin*	n.d.	1.215 (0.06)	n.d.	17.791 (0.83)	10.376 (0.155)	n.d.	7.342 (0.195)	0.827 (0.037)	0.151 (0.003)	8.798 (0.298)	3.147 (0.114)	1.219 (0.053)	0.811 (0.016)
**9**	404.25	403.25	*Naringenin-arabinoside*	2.003 (0.083)	4.232 (0.205)	17.671 (0.56)	10.611 (0.266)	1.473 (0.037)	1.774 (0.056)	5.769 (0.103)	5.056 (0.221)	2.132 (0.038)	6.761 (0.084)	4.016 (0.192)	22.161 (0.901)	1.173 (0.042)
**10**	418.23	417.23	*Kaempferol-arabinoside*	5.68 (0.243)	1.68 (0.08)	0.794 (0.021)	7.004 (0.075)	12.527 (0.424)	2.871 (0.032)	4.185 (0.067)	3.313 (0.06)	2.44 (0.056)	4.228 (0.126)	3.445 (0.164)	0.794 (0.019)	0.477 (0.013)
**11**	302.23	301.23	*Catechin-arabinoside*	3.477 (0.125)	1.321 (0.044)	0.897 (0.01)	5.378 (0.151)	1.99 (0.029)	n.d.	4.402 (0.204)	5.578 (0.121)	2.211 (0.073)	4.413 (0.164)	3.359 (0.039)	0.897 (0.027)	0.44 (0.008)
**12**	470.25	469.25	*Naringenin-glucoside*	4.686 (0.231)	4.365 (0.165)	14.706 (0.162)	10.122 (0.158)	9.976 (0.409)	6.018 (0.293)	9.587 (0.274)	12.757 (0.28)	8.686 (0.127)	3.229 (0.074)	9.197 (0.27)	1.446 (0.05)	1.05 (0.03)
**13**	316.23	315.23	*Catechin-rhamnoside*	1.635 (0.074)	1.991 (0.054)	4.299 (0.085)	2.449 (0.108)	2.398 (0.045)	2.121 (0.093)	3.567 (0.058)	4.077 (0.095)	2.71 (0.066)	0.7 (0.023)	2.426 (0.084)	0.464 (0.006)	0.308 (0.005)
**14**	450.25	449.25	*Dihydrokaempferol-glucoside*	1.694 (0.047)	1.954 (0.046)	8.217 (0.102)	7.923 (0.384)	3.426 (0.042)	1.997 (0.068)	9.927 (0.431)	5.466 (0.228)	2.901 (0.104)	8.807 (0.402)	6.523 (0.163)	0.401 (0.012)	0.78 (0.027)
**15**	332.36	331.36	*Catechin-glucoside*	n.d.	n.d.	2.139 (0.057)	7.927 (0.339)	6.286 (0.254)	n.d.	4.654 (0.084)	5.198 (0.08)	n.d.	4.846 (0.151)	3.381 (0.155)	2.45 (0.092)	0.555 (0.023)
**16**	448.25	447.25	*Kaempferol-glucuronide*	n.d.	18.246 (0.422)	9.909 (0.223)	34.671 (1.177)	36.72 (1.796)	n.d.	34.309 (1.322)	36.484 (0.786)	18.101 (0.392)	30.281 (0.453)	27.591 (0.676)	11.347 (0.255)	4.202 (0.108)
**17**	464.23	463.23	*Quercetin-glucoside*	n.d.	5.419 (0.072)	5.807 (0.113)	19.853 (0.317)	22.352 (1.045)	n.d.	13.488 (0.307)	14.465 (0.515)	4.317 (0.143)	15.82 (0.366)	14.242 (0.142)	6.65 (0.073)	1.663 (0.021)
**18**	478.26	477.26	*Isohamnetin-glucoside*	n.d.	4.27 (0.134)	15.15 (0.306)	13.615 (0.317)	6.535 (0.248)	n.d.	9.2 (0.288)	18.417 (0.698)	4.022 (0.092)	5.43 (0.264)	9.817 (0.395)	17.348 (0.738)	1.266 (0.035)
**19**	480.23	479.23	*Myricetin-glucoside*	n.d.	20.414 (0.83)	31.778 (1.351)	73.118 (3.655)	41.32 (2.048)	n.d.	31.774 (0.743)	36.544 (1.156)	12.307 (0.339)	30.727 (0.791)	37.513 (0.709)	36.387 (1.404)	2.965 (0.137)
**20**	482.25	481.25	*Dihydromyricetin-glucoside*	1.983 (0.082)	2.749 (0.058)	4.661 (0.094)	2.95 (0.03)	2.788 (0.118)	2.374 (0.061)	4.648 (0.182)	2.864 (0.041)	2.727 (0.07)	3.139 (0.06)	2.743 (0.098)	2.23 (0.037)	0.484 (0.006)
**21**	548.25	547.25	*Naringenin-sambubioside*	0.986 (0.046)	4.417 (0.153)	2.838 (0.072)	1.198 (0.026)	6.978 (0.218)	0.855 (0.018)	2.773 (0.114)	21.56 (0.68)	4.845 (0.067)	5.589 (0.242)	10.782 (0.519)	0.396 (0.015)	0.963 (0.024)
**22**	580.25	579.25	*Naringenin-rutinoside*	n.d.	n.d.	0.526 (0.019)	n.d.	n.d.	n.d.	1.024 (0.039)	0.769 (0.031)	n.d.	0.681 (0.021)	0.41 (0.018)	0.188 (0.004)	0.041 (0.001)
**23**	564.25	563.25	*Dihydrokaempferol-sambubioside*	n.d.	n.d.	0.559 (0.006)	n.d.	n.d.	n.d.	0.531 (0.009)	n.d.	n.d.	n.d.	n.d.	0.216 (0.008)	0.015 (0.001)
**24**	446.22	445.22	*Catechin-sambubioside*	n.d.	n.d.	0.706 (0.015)	n.d.	n.d.	n.d.	0.907 (0.025)	n.d.	n.d.	n.d.	n.d.	0.274 (0.003)	0.019 (0.001)
**25**	594.23	593.23	*Kaempferol-rutinoside*	0.346 (0.016)	1.959 (0.072)	0.659 (0.018)	3.821 (0.174)	3.255 (0.084)	0.742 (0.02)	2.924 (0.063)	4.134 (0.122)	0.298 (0.006)	2.219 (0.067)	3.06 (0.052)	0.787 (0.025)	0.225 (0.003)
**26**	596.25	595.25	*Dihydrokaempferol-rutinoside*	n.d.	0.969 (0.018)	5.266 (0.084)	1.59 (0.043)	11.411 (0.371)	n.d.	2.812 (0.076)	35.42 (1.745)	1.287 (0.042)	0.659 (0.023)	19.492 (0.411)	n.d.	0.751 (0.032)
**27**	610.23	609.23	*Quercetin-rutinoside*	0.934 (0.026)	0.8 (0.014)	0.909 (0.014)	5.308 (0.076)	1.48 (0.032)	1.68 (0.017)	1.299 (0.044)	1.76 (0.055)	1.318 (0.064)	1.507 (0.052)	1.187 (0.036)	1.029 (0.026)	0.188 (0.008)
**28**	612.25	611.25	*Dihydroquercetin-rutinoside*	0.738 (0.028)	1.267 (0.054)	1.31 (0.026)	1.736 (0.035)	0.524 (0.021)	1.343 (0.035)	3.274 (0.111)	3.369 (0.043)	1.081 (0.014)	4.31 (0.202)	4.161 (0.087)	1.03 (0.039)	0.209 (0.008)
**29**	484.35	483.35	*Catechin-diglucoside*	2.759 (0.085)	2.145 (0.081)	9.014 (0.417)	4.812 (0.22)	3.729 (0.089)	n.d.	1.425 (0.015)	6.994 (0.158)	4.788 (0.176)	1.433 (0.033)	2.054 (0.047)	3.536 (0.06)	0.41 (0.019)
**30**	624.26	623.26	*Isohamnetin-rutinoside*	1.5 (0.067)	n.d.	n.d.	1.649 (0.056)	1.336 (0.034)	n.d.	1.692 (0.041)	2.295 (0.102)	n.d.	1.481 (0.059)	1.567 (0.072)	n.d.	0.181 (0.006)
**31**	626.23	625.23	*Myricetin-rutinoside*	2.662 (0.077)	4.128 (0.047)	5.102 (0.096)	3.745 (0.12)	6.72 (0.296)	1.828 (0.044)	1.453 (0.031)	10.1 (0.149)	5.42 (0.108)	1.048 (0.035)	2.79 (0.099)	0.287 (0.011)	0.649 (0.023)
**32**	628.25	627.25	*Dihydromyricetin-rutinoside*	1.256 (0.057)	0.898 (0.04)	1.904 (0.069)	1.251 (0.044)	1.615 (0.024)	0.838 (0.021)	1.002 (0.043)	1.533 (0.069)	1.539 (0.016)	0.492 (0.011)	0.598 (0.009)	2.937 (0.07)	0.197 (0.008)
**33**	642.23	641.23	*Myricetin-diglucoside*	1.725 (0.036)	0.731 (0.016)	1.498 (0.019)	1.644 (0.019)	2.425 (0.029)	1.288 (0.039)	0.783 (0.032)	14.808 (0.382)	0.558 (0.009)	0.257 (0.005)	13.96 (0.469)	0.585 (0.025)	0.342 (0.005)
**34**	644.25	*643.25*	*Dihydromyricetin-diglucoside*	1.538 (0.024)	0.328 (0.011)	n.d.	n.d.	n.d.	n.d.	1.25 (0.032)	1.885 (0.034)	2.471 (0.11)	0.804 (0.019)	22.201 (0.947)	n.d.	0.354 (0.016)

Supporting Table 2: Qualitative and quantitative chemical analysis of N-containing compounds extracted from *G. simplicifolia* seeds identified by HPLC–ESI-MS/MS analysis. Table reports Molecular Weight (MW), ionized mass (m/z), chemical name and quantification in the different extracts. Data are expressed as µg per 100 g of FW. Data are expressed as mg per g of FW.

#	MW	m/z		Soxhlet			Acetone Maceration			Ethanol Maceration			Methanol Maceration			MAE
				Acetone	Ethanol	Methanol	50% (v/v)	70% (v/v)	95% (v/v)	50% (v/v)	70% (v/v)	95% (v/v)	50% (v/v)	70% (v/v)	95% (v/v)	
**35**	162.12	161.12	*1H-indole-3-carboxylic acid*	0.003 (0.001)	0.008 (0.001)	0.002 (0.001)	0.039 (0.001)	0.027 (0.001)	0.018 (0.001)	0.033 (0.001)	0.041 (0.001)	0.005 (0.001)	0.038 (0.001)	0.044 (0.001)	0.022 (0.001)	0.004 (0.001)
**36**	177.36	176.36	*5-hydroxytryptamine*	0.605 (0.016)	0.298 (0.01)	0.114 (0.001)	0.106 (0.001)	0.507 (0.015)	0.717 (0.007)	0.193 (0.005)	0.597 (0.009)	0.502 (0.01)	0.471 (0.005)	0.828 (0.031)	1.039 (0.018)	0.076 (0.002)
**37**	178.11	177.11	*5-Hydroxy-3-(2-hydroxyethyl)-indole*	0.347 (0.012)	0.143 (0.005)	0.148 (0.002)	1.229 (0.012)	0.459 (0.013)	0.327 (0.012)	0.22 (0.004)	0.168 (0.002)	0.285 (0.003)	n.d.	n.d.	n.d.	0.076 (0.002)
**38**	221.11	220.11	*5-hydroxytryptophan*	0.657 (0.019)	1.661 (0.06)	2.41 (0.081)	113.145 (2.438)	37.575 (0.551)	11.343 (0.287)	149.2 (1.415)	79.159 (1.506)	16.384 (0.308)	133.792 (2.927)	54.594 (1.66)	22.298 (0.51)	11.877 (0.236)
**39**	229.28	228.28	*3-Carboxy-6-hydroxy-β-carboline*	0.247 (0.008)	0.504 (0.006)	0.825 (0.025)	1.328 (0.018)	0.288 (0.007)	0.148 (0.004)	0.876 (0.015)	1.301 (0.029)	0.256 (0.006)	0.652 (0.018)	1.093 (0.035)	0.119 (0.001)	0.098 (0.001)
**40**	24616	24615	*Hyrtioerectine B*	0.945 (0.033)	1.189 (0.032)	0.769 (0.025)	5.332 (0.19)	2.207 (0.045)	0.937 (0.022)	4.977 (0.06)	3.491 (0.071)	1.016 (0.032)	3.07 (0.054)	3.372 (0.062)	1.006 (0.015)	0.331 (0.003)
**41**	330.32	329.32	*Griffonin*	0.068 (0.001)	0.146 (0.001)	0.242 (0.008)	0.394 (0.015)	0.08 (0.001)	0.038 (0.001)	0.258 (0.003)	0.386 (0.011)	0.071 (0.002)	0.19 (0.004)	0.323 (0.009)	0.029 (0.001)	0.023 (0.001)
**42**	344.05	343.05	*Hyrtiosulawesine*	0.071 (0.001)	0.145 (0.005)	0.112 (0.003)	0.466 (0.013)	0.335 (0.013)	0.221 (0.002)	0.436 (0.015)	0.478 (0.01)	0.134 (0.002)	0.487 (0.016)	0.531 (0.01)	0.282 (0.009)	0.052 (0.001)
**43**	235.21	234.21	*Tryptophan-4,5-dione*	5.138 (0.029)	5.397 (0.151)	5.468 (0.217)	1.277 (0.037)	1.15 (0.035)	0.518 (0.013)	1.508 (0.024)	1.652 (0.098)	0.57 (0.011)	1.758 (0.061)	1.697 (0.068)	0.921 (0.013)	0.455 (0.024)
